# A Social-Ecological Framework to Understand Barriers to HIV Clinic Attendance in Nakivale Refugee Settlement in Uganda: a Qualitative Study

**DOI:** 10.1007/s10461-020-03102-x

**Published:** 2020-12-02

**Authors:** Kelli N. O’Laughlin, Kelsy Greenwald, Sarah K. Rahman, Zikama M. Faustin, Scholastic Ashaba, Alexander C. Tsai, Norma C. Ware, Andrew Kambugu, Ingrid V. Bassett

**Affiliations:** 1 Departments of Emergency Medicine and Global Health, University of Washington, 1959 NE Pacific St, Seattle, WA 98195 USA; 2 Harvard Affiliated Emergency Medicine Residency, Boston, MA USA; 3 Wesleyan University, Middletown, CT USA; 4 Bugema University, Kasese Campus, Kampala, Uganda; 5 Mbarara University of Science and Technology, Mbarara, Uganda; 6 Center for Global Health, Massachusetts General Hospital, Boston, MA USA; 7 Harvard Medical School, Boston, MA USA; 8 Department of Medicine, Brigham and Women’s Hospital, Boston, MA USA; 9 Makerere University, Infectious Diseases Institute, Kampala, Uganda; 10 Department of Medicine, Massachusetts General Hospital, Boston, MA USA

**Keywords:** Refugee, HIV, Qualitative, Linkage

## Abstract

The social-ecological model proposes that efforts to modify health behaviors are influenced by constraints and facilitators at multiple levels. We conducted semi-structured interviews with 47 clients in HIV care and 8 HIV clinic staff to explore how such constraints and facilitators (individual, social environment, physical environment, and policies) affect engaging in HIV clinical care in Nakivale Refugee Settlement in Uganda. Thematic analysis revealed that participants were motivated to attend the HIV clinic because of the perceived quality of services and the belief that antiretroviral therapy improves health. Barriers to clinic attendance included distance, cost, unemployment, and climate. Those that disclosed their status had help in overcoming barriers to HIV care. Nondisclosure and stigma disrupted community support in overcoming these obstacles. Interventions to facilitate safe disclosure, mobilize social support, and provide more flexible HIV services may help overcome barriers to HIV care in this setting.

## Introduction

In 2001, the General Assembly of the United Nations adopted the Declaration of Commitment on HIV/ AIDS, “recognizing that populations destabilized by armed conflict, humanitarian emergencies and natural disasters, including refugees, internally displaced persons, and in particular women and children, are at increased risk of exposure to HIV infection.” [1] There are 25 million refugees worldwide [2]. Uganda alone hosts over 1.4 million refugees, [2] and is the largest refugee hosting country in Africa [3]. Refugees are at risk of exposure to HIV and face unique barriers to receiving and maintaining antiretroviral therapy (ART). Specific programs should be designed to overcome these distinctive barriers to HIV clinical care faced by refugee populations.

Scaling up HIV client-initiated testing and counseling has been a top priority of the United Nations High Commissioner for Refugees (UNHCR), the World Health Organization (WHO), and the Joint United Nations Programme on HIV/AIDS (UNAIDS) for more than a decade [4]. Like many refugee settings, Nakivale Refugee Settlement in Uganda has clinics that offer free HIV testing and antiretroviral medication, which can be accessed by both refugees and nationals. Remaining barriers to HIV testing in Nakivale include individuals’ focus on urgent tasks aimed at meeting daily survival needs such as accessing food, maintaining shelter and ensuring their own safety [5]. While health care workers have been trained to provide confidential and voluntary testing in UNHCR-run camps, there continues to be difficulty in translating increased testing to increased HIV treatment and care [6]. Although some of these barriers are shared among others in Uganda living with HIV, refugees are uniquely vulnerable to food insecurity, drug stockouts, difficulty accessing clinic when ill, and violence and unrest [7]. As HIV testing becomes more available in refugee settings, facilitating HIV clinic attendance and engagement in care is the next step in achieving viral suppression and preventing the spread of the disease.

We sought to use the social-ecological model as a framework to assess health behavior in refugee settlements and barriers and facilitators to engagement in HIV care. We aimed to identify potential modifiers of those barriers and facilitators in a humanitarian setting. The social-ecological model proposes that individual health has multiple individual, physical, social, and regulatory influences [8–11]. Efforts to modify health behaviors must take these multiple levels of constraints into account [12–15]. Our objective was to evaluate the individual, physical, social, and regulatory factors influencing HIV care utilization in Nakivale Refugee Settlement in Uganda. Using this framework, we sought to better understand why refugees make the health decisions that they do and determine how best to motivate change in this environment.

## Methods

### Study Site

Nakivale Refugee Settlement is located in a rural region of southwestern Uganda, is 71 square miles and is home to approximately 100,000 refugees from the Democratic Republic of the Congo, Rwanda, Somalia, Burundi, Eritrea, Ethiopia, Sudan, South Sudan, Kenya, and Liberia [16]. Given HIV prevalence among refugee communities are dependent upon the HIV prevalence pre-conflict, the HIV prevalence among the surrounding host community, exposure to violence during conflict and flight and the level of interaction between the refugee and host community, there is a wide variety of sexual risk and HIV prevalence among refugees in Nakivale [17]. First, the countries of origin of the refugees living in Nakivale have a lower HIV prevalence than Uganda, thus increasing the risk of acquiring HIV for refugees that have been settled longer and have had more interactions with the Ugandan population [18]. Second, refugees from different countries of origin faced varying levels of exposure to violence and rape as a weapon of war. Third, even within the refugee population, there are those such as sex workers who face increased risk of contracting HIV. The local economy consists primarily of activities in animal husbandry, subsistence farming, and petty trading; food and water insecurity are fairly common [19–21]. At Nakivale Health Center, a clinic located within Nakivale Refugee Settlement, both refugees and Ugandan nationals can access HIV testing and ART free of charge. As of September 2017, in the combined Nakivale Refugee Settlement and neighboring Oruchinga Refugee Settlement, HIV prevalence among those testing through the health facilities was 2.3%, and 3,472 HIV clients were on ART, of which 37% were refugees and 63% were nationals [22]. A prior study in Nakivale found that of those that underwent routine HIV testing (N = 4207) and were newly diagnosed with HIV (N = 188), only 54% linked to HIV clinical care and only 6% initiated ART [6, 23, 24].

### Qualitative Protocol

Research assistants recruited participants for this study by offering group information sessions about HIV and by offering HIV testing twice daily for those waiting in the Outpatient Department. After giving written consent, those willing to participate in the HIV testing research verbally completed a demographic questionnaire, including questions regarding date of birth, gender, country of origin, marital status, education level, religion, and distance to clinic which was read to them by a research assistant with information directly entered into an electronic database. The clients then individually accompanied the counselor to a private testing area where they underwent HIV testing and were provided results. Each client was offered a post test counseling session and information regarding HIV medication and services at the health center. Willing participants newly diagnosed with HIV were invited to interview 90 days after HIV testing. Everyone invited to participate in the study was willing to participate. Research assistants also recruited staff members who were interviewed without undergoing HIV testing. Inclusion criteria for the client qualitative interviews were: (1) ≥ 18 years of age, (2) not previously diagnosed with HIV, (3) have not participated in this study within the last three months, (4) willing to have future lab tests and clinic attendance tracked for study purposes, (5) able to understand the counseling session and what is involved in the study, and (6) willing and able to give informed consent. There were no specific exclusion criteria, other than not meeting inclusion criteria.

Between August 2, 2016 and March 13, 2018, semi-structured interviews were conducted in-person at a location specified by the participant. Most interviews were conducted at the Nakivale Health Center. Interviews lasted approximately 45 min. Interviews were conducted in English, Kinyarwanda, Runyankore, or Kiswahili by refugee research assistants living in the region trained in qualitative methods; one was male and three were female.

Semi-structured interviews were conducted by research assistants trained in qualitative techniques. The interviews were open ended to ensure both that core topics were covered and that unanticipated themes could arise [25, 26]. Following a social ecological model framework [5–8], the interviews were structured to explore the following levels of influence to engagement in HIV clinical care: individual, social environment, physical environment, and regulatory policies. Open-ended questions such as *“Was there anything that made it easier or more difficult for you to come to clinic?”*, *“In what ways does the job make it easier or harder?”*, and *“Do the ways people in your family or community think and talk about HIV impact your clinic attendance? How?”* were posed to refugees and nationals to evaluate these levels of influence. Additionally, ideas on how to improve linkage to clinic were investigated.

### Data Analysis

Interviews were audio-recorded and were transcribed directly into English. We used Dedoose**™** qualitative software to organize the data [27]. One investigator reviewed the transcripts for relevant content and used that content to draft the codebook. A second investigator read three transcripts; the two investigators worked together to revise the codebook. To improve accuracy, a third investigator drafted a separate codebook based on reviewing the same three transcripts without input from the other analysts and without viewing their codebook. The three investigators compared their codebooks and reached a consensus on a revised codebook. Major categories included: disclosure of HIV status, distance to clinic, money/cost, perceptions regarding health clinic services provided by the health center, facilitators and barriers to clinic attendance, stigma, and competing needs. Two investigators then coded 3 transcripts aloud while further refining the codebook by merging some codes and deleting others. The initial inter-rater reliability when these investigators coded 5 transcripts was an initial pooled Cohen’s kappa of 0.66. The investigators discussed discrepant codes, improving to a final Cohen’s kappa of 1.00 (good–excellent). The three investigators then proceeded with coding all the transcripts using the final codebook [28–30]. Once the transcripts were coded, the three investigators re-read the transcripts and excerpts within each code to identify common themes. The first two investigators then worked to organize these themes into categories that adequately reflected facilitators and barriers to HIV clinic.

## Results

### Study Participants

Interviews were conducted with a total of 47 clients and 8 staff members. The average age of clients was 32 years. Client participants were predominantly male (57%, n = 27). The majority of clients were Ugandan nationals (70%, n = 32) vs. refugees (30%, n = 14). Of the Ugandan nationals, 21 (66%) were male and 11 (34%) were female. Of the refugees, 5 (36%) were male and 9 (64%) were female. Client participants’ countries of origin included the Democratic Republic of the Congo (15%, n = 7), Rwanda (8%, n = 4), Burundi (8%, n = 4), and Uganda (67%, n = 31). The majority were married (60%, n = 28), Christian (66%, n = 31), primarily spoke Runyankole (64%, n = 29), and had completed some primary school (57%, n = 27). The average number of years living in Nakivale was 9 years, including time reported by both refugees and Ugandan nationals.

Clinic staff participants were majority male (75%, n = 6), married (63%, n = 5), Ugandan (88% n = 7), and had a diploma or completed a certificate program (75%, n = 6). The average age of clinic staff was 33 years.

### Qualitative Results

Refugees and Ugandan nationals were motivated to attend HIV clinic, but were hindered by multiple daily challenges. Those who were willing to disclose their HIV status often benefited from social support, which offered practical assistance such as money or transportation to overcome these structural barriers. However, stigma often prevented such disclosure, leaving participants isolated and unable to engage in HIV care.

#### Motivated to Come to Clinic

Refugee and Ugandan participants were motivated to attend the HIV clinic because of perceived quality of clinic services, specifically how counselors taught the clients about HIV, dispelled myths and advised them to take their medication. Clients also felt that improvement in their health was due to ART, and therefore wanted to continue taking their medication.

##### Clinic Health Services (Regulatory)

The clinic offered a variety of services to their clients, including counseling, medication, mental health services, and support. Often the counseling and encouragement became a key factor in influencing clients to return to clinic. Participants felt staff members helped “*teach how to handle the situation or how to live a positive life after being HIV infected (female refugee)*.” Staff members agreed that often “*teaching and counseling is more useful than getting drugs because it is more important to show them that there is no problem to be infected (female refugee)*.”

##### Medication Effects (Individual)

After undergoing counseling at the clinic, refugees and Ugandan nationals believed that ART improved their health. They would feel healthier after coming to clinic, and linked their current health status to their clinic attendance and medication adherence. According to one refugee, *“I know many people who died with AIDS, my parents included. But for them there were no drugs to help them during their times, so I have to take drugs in order to get my life prolonged because now since I began drugs I feel fine and strong (female refugee).”*

#### Barriers to HIV Clinic Care

Despite significant motivation, refugees and Ugandan nationals faced many barriers to attending HIV clinic after being diagnosed with HIV.

##### Transport/Distance (Physical)

Many clients described walking distances of 5–6 h to reach the health facility, often leaving before daylight to make it to clinic on time and to have time to return home. Some participants were deterred from attending closer clinics, “*though Mbare is near and has HIV services but I can’t go there as sometimes they don’t have drugs (female Ugandan national)*.” Walking long distances and leaving home for a full day is especially difficult for “*those who stay alone, or [for those] with children like a mother, a widow with her small children and she is also sick becomes very hard to walk very long distance coming to a health facility (staff member)*.”

##### Money/Cost (Regulatory)

Although clinic visits and ART are free, transportation “*like getting a boda boda [hired motorcycle taxi] is always hard because it all requires money. More to that the distance is too long where by you cannot move by foot (female Ugandan national)*.” At other times, the clients felt too unwell to walk such long distances, and had to hire a boda boda even for distances that would have been 1–2 h on foot. In order to raise money, clients were forced to “*look for casual labor and dig as I don’t have elsewhere to get money from my husband can’t allow me to at least cut a fresh banana to sell but tells me that’s your own business so I try my level best and make sure I do whatever it takes to get the transport (female Ugandan national)*.” Women in particular struggled. Because men often controlled the money in the household, and at times refused to allow their wives to spend it on transportation to HIV clinic, wives felt they must “*hide [the money from their husbands] as women do and after selling I get the money that can help me secretly mostly to come and get drugs (female Ugandan national)*.” Those without husbands were not better off, “*I am widow so I have to struggle for it. I have to work for food to feed my child and look for money to bring me (female refugee)*.”

##### No Employment (Regulatory)

Although some clients worked and could pay for transportation, most had difficulty finding employment. For some, their health prohibited difficult labor – “*transport has been a problem because I struggle to get it yet I do not have enough energy to work (male Ugandan national).*” Others struggled with employment because most opportunities were outside of the settlement – “*There [are] no jobs in Nakivale so I get out to look for employment (male Ugandan national)*.” Unlike other refugees, most refugees living with HIV could not work outside the settlement for an extended period of time. Instead they and Ugandan nationals had to return to clinic every month for their medication. “*I feel like I can go and look for the job in [far away town], but then, I start asking myself what I will do when my drugs get finished (male, unidentified whether refugee or Ugandan national)*.” Once they work far from the clinic, “*getting transport for every appointment becomes a problem it impacts my attendance (male Ugandan national)*.”

##### Climate and Season (Physical)

Refugees and Ugandan nationals in Nakivale were particularly vulnerable to climate changes. At times, extreme rain or heat simply made it difficult to walk for six hours to clinic, “*you fail to proceed as no way to pass because of the rain (female Ugandan national).”* Because of food distribution polices in the Settlement, most refugees also provided food for themselves and their family through farming and digging. If the weather prohibited growth, they often had no other way to feed themselves, forfeiting what little money they had for food instead of transportation. Food insecurity offered another barrier to HIV clinic, as food often took priority over attending clinic. *“Some seasons make people move from their homes to go and struggle and look for food and money like when it is dry season you find there is no maize flour, no soya flour, and you need to eat and when you go far away you may miss your appointment date because you even lack transport to the HIV clinic to pick your drugs (male Ugandan national).”*

#### Disclosure

##### Overcoming Barriers Through Disclosure (Social)

Life in Nakivale is communal and social. Refugee and Ugandan nationals more easily overcame barriers to engagement in HIV clinic when they had help [31]. For instance, friends and family provided money for transport or child care while the client traveled to clinic. When a friend or family member is aware of a client's HIV status, “*she encourages me and also remains with my child when I am coming here [HIV clinic] (female Ugandan national),”* and *“they give some money to help me on the way and when I go back home I find when food is ready (male Ugandan national).”* Meeting other clients living with HIV normalized HIV, offered emotional support, and fostered hope, *“coming to the HIV clinic helps me to meet others who have the same case and I don’t feel alone I get encouraged (female refugee)*.” They can often rely on each other for moral and tactical support, *“We all come together to pick drugs and always remind each other when to come to pick drugs (female refugee).”*

Relying on assistance from others was predicated on disclosing HIV status to friends or family who could help, “*There are those whom I trusted and I told them and they also helped in counseling (male Ugandan national)*.” Often clients were only comfortable disclosing their status to family, clinic counselors or other individuals living with HIV – “*I talk to my family members. I always talk to the health workers (male Ugandan national).”* Many refugees and Ugandan nationals in Nakivale are intentionally guarded with regards to disclosing their HIV status. Some choose to only disclose to necessary people so that they can access the support they require or the support they anticipate they may require to meet their basic needs. *“No one knows my HIV status [ex]cept my children whom I told that I am HIV positive, so that when they see me seriously sick they could take me at the clinic. But can’t tell everyone I am HIV positive (female Ugandan national).”*

##### Stigma and Nondisclosure (Social)

Others refused to share their status because of stigma and the fear that they will then lose social support in the other facets of their life, such as collecting food. Because of the discrimination faced by individuals living with HIV faced, many clients chose not to disclose their status to friends or family, in the hopes that by hiding their status they could continue to access social support essential for survival. *“When you are HIV infected person you don’t tell everyone your status because you will be isolated as no one will associate with you as before (female refugee).”* Without this social support, clients often did not have their basic needs met, “*People who could help me stay far from me. I can’t get someone to prepare for me this kashera or cooking water for drinking (male Ugandan national)*.” The clients feared the community they relied on would reject them, “*There is no help I cater for myself (male Ugandan national)*.” They also could not access the HIV clinic or their medication,*“I spent three days without taking drugs since I was sick and I didn’t have drugs because I couldn’t come here and there is no one who came to pick my drugs because I had not even told anybody (female Ugandan national).”* The social isolation itself was demoralizing, *“You know with HIV, someone reaches at a time when he or she becomes helpless they always feel isolated because they know that they cannot be helped by anybody (staff member),” a*nd could ultimately affect clients’ perception of their own fight against HIV, *“You find that people are always talking about you, you become stigmatized and sometimes you refuse taking your drugs (female Ugandan national)*.”

**Fig. 1:**
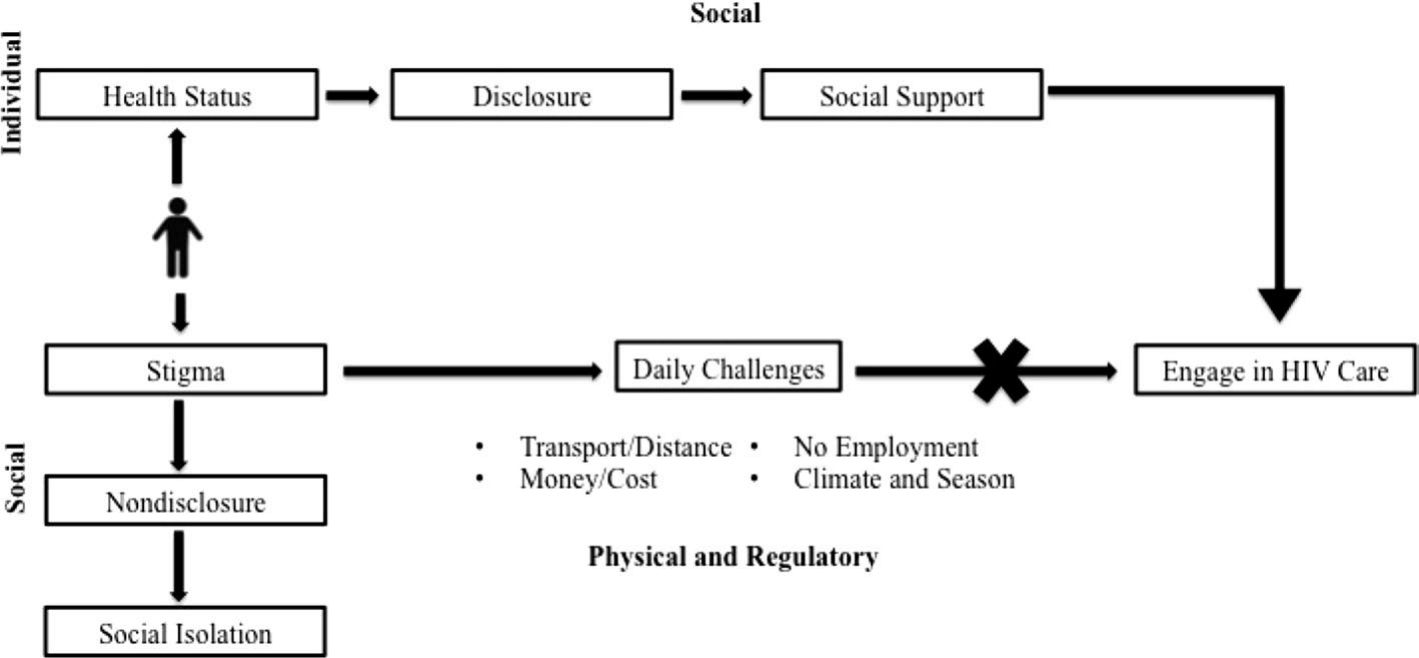
Conceptual Framework of factors that assist and hinder engagement in HIV care in a refugee settlement

As illustrated below (Fig. 1), clients accessing HIV medication at the Nakivale Health Center faced many barriers and daily challenges, including transportation, money, lack of employment, and climate. Many clients could not afford to pay for transportation, were restricted in traveling for work, and were particularly vulnerable to climate changes that resulted in food shortages. Those that were willing to disclose their status to trusted friends and family members had help in overcoming these daily challenges. Clients who could rely on social support were often given money, transportation, and food. However, clients living with HIV in Nakivale Refugee Settlement often chose not to disclose their seropositivity to friends and family or would only disclose to a select few because of the stigma associated with HIV. Nondisclosure led to social isolation, making it difficult for many to rely on community support to overcome obstacles to effectively engage in HIV clinical care.

## Discussion

Adapting the social-ecological model for the refugee context, we identified four levels of influence on health behavior as it related to engagement in HIV clinical care. First, on the individual level, a client's physical health status and emotional well-being affected his or her ability to attend clinic. For these clients who were able to attend clinic, the individual context was a motivator, or facilitator. Second, the physical context of the refugee settlement, the distance to clinic or the physical climate, presented its own barriers to engaging in HIV care. Third, policies and regulations, such as the need to farm for food or search for employment outside of the settlement, similarly hindered those restrained by the need to make monthly trips to the clinic [9, 10]. Fourth, the social context of the refugee settlement was the most complex, and offered both barriers and facilitators to engagement in HIV care. Much like other regions of Uganda [32, 33], Nakivale has a communal atmosphere, requiring social cohesion in order to meet daily survival needs and prioritize clinic attendance. Also, men in Nakivale often controlled money in the household; a female refugee often had to ask permission or raise money by herself in order to attend clinic. Because of the level of social cohesion in the settlement, clients were incentivized to disclose their status, which would allow them to partake in the many aspects of social support (i.e. borrowing money, rides) that would facilitate attending clinic. However, stigma and the fear of losing the social support necessary in other avenues of life led other clients to hide their status, resulting in further social isolation and creating more barriers to clinic. Using the social-ecological model allowed us to evaluate how these four milieus influenced ability to access HIV clinic in this humanitarian setting, and how we can target interventions to promote access.

Perceived stigma has been previously identified as an important obstacle to accessing HIV clinical care. A study in South Africa found that the fear of stigmatization was a deterrent to both HIV testing and treatment [34, 35]. Similarly, a study in northwestern Cameroon found the fear of disclosure, stigma, and discrimination were barriers to HIV testing [36]. Stigma has been found to inhibit disclosure of serostatus to others [37], thereby compromising the ability of people living with HIV to mobilize support from among those in their social networks [38–40]. These studies are also consistent with population-level evidence linking stigma to lack of HIV testing [41] and treatment non-adherence [42]. This study adds to the literature by showing the impact of stigma on HIV care engagement particularly in the humanitarian context.

Our qualitative data identified opportunities to promote HIV clinic attendance. When specifically asked which interventions would improve their ability to attend clinic, refugees and Ugandan nationals living with HIV consistently recommended assistance in transport [43–46], money, and food [47–49]; these were categories which some were fortunate enough to receive from their friends and family if they had disclosed their status, which many do not. Persistent teaching, de-stigmatizing HIV, and community outreach could significantly benefit refugees and Ugandan nationals living with HIV. As this will take time, in the interim, strategies should also focus on promoting social support among individuals living with HIV. For instance, to tackle the barrier of distance to clinic, clients with HIV could help pick up and distribute antiretroviral medications to each other. This strategy has been studied by Decroo et al*.* and Médecins Sans Frontières in Mozambique, which showed that patients who participated in community ART groups, and took turns collecting ART medications at clinic, had higher rates of retention in care [50–56].

This study must be interpreted in the context of certain limitations. This study did not include those who had stopped participating in clinical care. Therefore, insurmountable barriers that would have prohibited any engagement in clinical care may not have been explored. Barriers to reengaging in care were also not evaluated. Furthermore, the overall numbers of participants, once broken down by gender and Ugandan national vs. refugee, are too small to perform a detailed analysis by gender. Stigma was a significant barrier to attending HIV clinic. Fear of stigma and refusal to disclose HIV status limited clients’ abilities to attend clinic. However, more research is needed to quantify the push and pull of the potential social harm of stigma after HIV status disclosure and the need for social support [57, 58]. Stigma may discourage people diagnosed with HIV in refugee settlements to disclose their status. On the other hand, disclosure in a humanitarian setting may be higher risk given the reliance on social support to survive.

## Conclusion

Qualitative interviews with refugees and Ugandan nationals attending HIV clinic while living in Nakivale Refugee Settlement demonstrated barriers on the individual, social, physical, and regulatory levels. Disclosure of HIV status increased access to social support and facilitated engagement in HIV care. Expanding education programs may decrease stigma surrounding HIV. Interventions to facilitate safe HIV status disclosure, mobilize social support, and provide more flexible HIV services may help overcome barriers to HIV care in this setting.
